# Cryptorchidism in Children with Zika-Related Microcephaly

**DOI:** 10.4269/ajtmh.19-0753

**Published:** 2020-03-09

**Authors:** Rômulo A. L. de Vasconcelos, Ricardo A. A. Ximenes, Adriano A. Calado, Celina M. T. Martelli, Andreia V. Gonçalves, Elizabeth B. Brickley, Thalia V. B. de Araújo, Maria Angela Wanderley Rocha, Demócrito de B. Miranda-Filho

**Affiliations:** 1 Universidade de Pernambuco, Recife, Brazil;; 2 Universidade Federal de Pernambuco, Recife, Brazil;; 3 Instituto de Pesquisa Aggeu Magalhães, Fiocruz, Recife, Brazil;; 4 London School of Hygiene and Tropical Medicine, London, United Kingdom

## Abstract

The genitourinary tract was recently identified as a potential site of complications related to the congenital Zika syndrome (CZS). We provide the first report of a series of cryptorchidism cases in 3-year-old children with Zika-related microcephaly who underwent consultations between October 2018 and April 2019 as part of the follow-up of the children cohort of the Microcephaly Epidemic Research Group, Pernambuco, Brazil. Of the 22 males examined, eight (36.4%) presented with cryptorchidism. Among 14 undescended testis cases, 11 (78.6%) could be palpated in the inguinal region. Seven of the eight children had severe microcephaly. Conventional risk factors for cryptorchidism were relatively infrequent in these children. We hypothesize that cryptorchidism is an additional manifestation of CZS present in children with severe microcephaly. As in our cases, for most of the children, the testes were located in the inguinal region, and the possible mechanisms for cryptorchidism were gubernaculum disturbance or cremasteric abnormality.

Congenital Zika syndrome (CZS) is a unique pattern of anomalies and disabilities found in children infected with Zika virus (ZIKV) in utero. Although neurologic impairments, including morphological alterations – most notably, microcephaly – and radiologic anomalies, including brain calcifications, are among the most common features, it is increasingly recognized that CZS can be associated with abnormalities across a range of organ systems.^1^ It is now recognized children with CZS may have increased risk of neurogenic bladder.^2,3^ Nevertheless, the full range of urogenital complications remain inadequately investigated.

Here, we provide the first report of cryptorchidism cases in children with Zika-related microcephaly. Cryptorchidism is one of the most common congenital malformations of males occurring in about 1% of the boys at the end of the first year of life and is associated with testicular cancer, infertility, and hypogonadism.^4^ Although there is a known association between cryptorchidism and cerebral palsy, the link with ZIKV is limited to a single postmortem report of cryptorchidism in a neonate with microcephaly and other severe defects who died within 20 hours of birth.^5,6^

All parents/caregivers signed an informed consent form. This study was approved by the Ethical Committee (CAAE: 94544518.2.0000.5192).

This case series includes all 3-year-old male patients with Zika-related microcephaly who underwent consultations by a pediatric urologist between October 2018 and April 2019 as part of the follow-up of the children cohort of the Microcephaly Epidemic Research Group (MERG), Pernambuco, Brazil (Study Protocol: http:/www.cpqam.fiocruz.br/merg/). The criterion to refer to the pediatric urologist was exclusively the diagnosis of Zika-related microcephaly. The MERG is following approximately 200 children with microcephaly (i.e., head circumference of more than two SDs below the mean for their gender and gestational age) identified mainly during the microcephaly epidemic in the state of Pernambuco with clinical and/or radiological and/or laboratorial evidence of Zika exposure.^7,8^ During these evaluations, 22 microcephalic males (i.e., totaling 44 testicular units) were evaluated using the 2018 pediatric guidelines of the European Association of Urology.^9^ According to these guidelines, true cryptorchidism is defined as testis that cannot be brought to the scrotum during physical examination.

Data on potential risk factors for cryptorchidism^10^ were either retrieved from the MERG database (prematurity, birth weight, and delivery type) or obtained by telephone interview with the mothers (birth presentation, assisted reproduction, familial smoking, twinning, race, and family history of cryptorchidism). One mother was not available for telephone interview.

Of the 22 males with Zika-related microcephaly examined, eight (36.4%) presented with true cryptorchidism. Seven boys with retractile testis were not included. The other seven did not present cryptorchidism. The age at diagnosis of cryptorchidism ranged from 8 days to 46 months. Of the eight children, five had microcephaly at birth, one classified as moderate microcephaly (≤ −2 SD) and four as severe (≤ −3 SD). The other three children were diagnosed with severe microcephaly at the first evaluation. Of these eight children, one or both testis had failed to descend to the scrotum, were unable to be mobilized and maintained in their anatomical position, and/or were absent/undetectable on clinical examination. Of the cryptorchidism cases, six were bilateral, one was on the right, and one on the left (Table 1). Six of the children had palpable gonads in the inguinal region, which included six children with the testis on the right and five on the left. Two of the children had non-palpable testes, which were bilateral in one child and occurred only on the left in another. One of the children had previously undergone surgery at 2 years of age, and on that occasion, the testes were located in the inguinal region.

**Table 1:** Clinical characterization of a case series of cryptorchidism in eight male children with Zika-related microcephaly

Laterality of cases of cryptorchidism (*n* = 8)		
Bilateral	6	75.0%
Right only	1	12.5%
Left only	1	12.5%
Situation of testicular units with cryptorchidism (*n* = 14)		
Non-palpable on the right	1	7.1%
Non-palpable on the left	2	14.3%
Palpable on the right	6	42.9%
Palpable on the left	5	35.7%
Background of children with cryptorchidism		
Gestational age at delivery		
< 37 weeks	1	20.0%
≥ 37 weeks	4	80.0%
Missing	3	–
Weight at birth		
< 2,500 g	2	28.6%
≥ 2,500 g	5	71.4%
Missing	1	–
Delivery type		
Vaginal	4	57.1%
Caesarean	3	42.9%
Missing	1	–
Assisted reproduction		
Yes	0	0%
No	7	100.0%
Missing	1	–
Maternal smoking during pregnancy		
Yes	0	0%
No	7	100.0%
Missing	1	–
Paternal smoking during pregnancy		
Yes	1	16.7%
No	5	83.3%
Missing	2	–
Twinning		
Yes	1	14.3%
No	6	85.7%
Missing	1	–
Self-identified race/ethnicity		
Mixed	6	85.7%
Black	1	14.3%
Missing	1	–
Family history of cryptorchidism		
Yes	0	0%
No	7	100%
Missing	1	–
Microcephaly		
Moderate (≤ −2SD)	1	12.5%
Severe (≤ −3SD)	7	87.5%
Arthrogryposis		
Present	2	25%
Absent	6	75%
Muscular tone		
Normal	1	14.30%
Axial and appendicular hypotonia	1	14.30%
Axial and appendicular hypertonia	2	28.55%
Axial hypotonia and appendicular hypertonia	2	28.55%
Appendicular hypertonia	1	14.30%
Missing	1	–

Among the five children with cryptorchidism and available gestational age at birth information, only one was born less than 37 weeks. Four of seven with complete data on delivery mode were born vaginally with cephalic presentation. In the three children born by caesarean section, the mothers had not been informed by the obstetrics team of the reason for this option. With respect to the birth weight, two weighed less than 2,500 g at delivery, and the birth weight was unreported in one case. None of the children were reported to be born from pregnancies resulting from assisted reproduction. Of the families with available data, no mothers and only one father reported smoking during pregnancy. One child with cryptorchidism was reported to be a twin. In terms of the children’s race/ethnicity, six mothers classified their children as being of mixed ethnicity, and one identified her child as black. None of the families interviewed had a history of cryptorchidism.

One child was diagnosed as early as 8 days indicating a congenital cryptorchidism. Later diagnosis in the other children may have occurred either because of missed diagnosis or may represent cases of late ascended testis. Most of the testicular units in this series of cases were palpable in the inguinal region, and, therefore, had successfully concluded the phase of intra-abdominal descent. This finding suggests a lower probability of interference in the early stages of embryonic testicular development, which occurs between the fourth and 12th week of gestation or in the production and/or action of insulin-like 3 protein, responsible for the intra-abdominal descent phase. As in our cases, for most of the children, the testes were located in the inguinal region, a possible explanation is that the mechanism that produced cryptorchidism was a gubernaculum disturbance because it is responsible, under androgenic action, for transferring the testicles to the scrotum through the peritoneal–vaginal conduit until the 35th week of gestation.^9,11^ Genitofemoral nerve issues, which ultimately affect gubernacular function, may also be associated with mechanisms of cryptorchidism. The finding of cryptorchidism with three impalpable gonadal units in two boys still requires surgical exploration to confirm the true nature of their involvement, which may be related to agenesis, congenital atrophy (vanishing testis), or ectopic testis. None of the children presented with genital anatomical abnormalities, suggesting that the cases in question are not related to specific urological syndromes. Non-syndromic cryptorchidism in humans is multifactorial and mediated by an intricate neurohormonal imbalance.^12^ The children in this series presented with severe microcephaly associated with significant impairment of the central nervous system, similar to that which occurs with cerebral palsy. Although an association between cerebral palsy and cryptorchidism has been described, the underlying mechanisms remain unclear.^5,13^ One possible mechanism for the findings could be alterations to the cremaster muscle, which has been especially suggested in cases of ascended testis. In these cases, the cremasteric reflex, influenced by neurological impairment, could be responsible for late ascension of gonads, but gubernacular abnormal insertion has been suggested as a cause too.^5,14^

In our case series, traditional risk factors^10^ do not seem to explain the presence of cryptorchidism. We observed just two cases of low birth weight and one case of prematurity. All vaginal deliveries were of cephalic presentation. Among the cases delivered by caesarean section, it was not possible to obtain the data that led the medical team to opt for the caesarean section, and it is possible that a non-cephalic presentation could have been the reason for such a recommendation. We also note that despite public policies to encourage transvaginal birth, the caesarean rate in Brazil is high, so it is plausible that the delivery mode was not medically indicated. In the present series, no pregnancy had resulted from assisted reproduction. Only one case of paternal smoking was reported in the children. Only one case of twinning was reported. Regarding ethnicity, six children were identified by their mothers as being of mixed ethnicity and one as black, so any ethnicity-related patterns were not discernible. We also not had a reported case of family history of cryptorchidism.

Although this investigation had limitations because of the small sample size and the absence of surgical exploration, this study is of public health significance in that it provides the first description of cryptorchidism in a series of children with CZS. It is not likely that the very high rate (30%) of cryptorchidism found is due to selection bias. Even if all children with microcephaly were not evaluated by a pediatric urologist, referral to this specialist was not based on any clinical finding; therefore, we will not expect that the frequency of cryptorchidism will be different in those not yet examined. In addition, eight of 91 (total number of microcephalic boys followed by the MERG) is still a high frequency.

Although conventional risk factors for cryptorchidism were relatively infrequent in these children, all were affected by severe impairment of the central nervous system. Therefore, we hypothesize that cryptorchidism is an additional manifestation of CZS present in children with severe microcephaly. We suggest that the observed cases may be associated with gubernacular and cremasteric abnormalities, although surgical exploration will be essential for a better understanding of possible mechanisms. Because of the greater risk of future complications such as functional impairment of the gonads, as well as cancer, male children with Zika-related microcephaly should also undergo routine urogenital assessment. Further studies should be performed to better characterize this new clinical feature associated with CZS and to clarify the mechanisms leading to cryptorchidism in these children.
